# CRISPRi screening identifies PIKfyve as a co‐therapeutic target for obinutuzumab

**DOI:** 10.1002/ctm2.70333

**Published:** 2025-05-07

**Authors:** Yerim Kim, Jinkyung Oh, Jeong Ryeol Kim, Donghyuk Lee, Joo Young Kim

**Affiliations:** ^1^ Department of Pharmacology and Brain Korea 21 PLUS Project for Medical Science Yonsei University College of Medicine Seoul Republic of Korea; ^2^ Woo Choo Lee Institute for Precision Drug Development Seoul Republic of Korea

1

Dear Editor:

Combining monoclonal antibodies with targeted agents is a promising yet mechanistically underexplored strategy for B‐cell lymphoma therapy.^1^ Through a CRISPR interference (CRISPRi) screen, we identify PIKfyve as a suppressor of obinutuzumab‐induced lysosomal membrane permeabilisation (LMP) and direct cell death (DCD), highlighting its potential as a combinatorial therapeutic target.

Obinutuzumab (OBI), a next‐generation anti‐CD20 monoclonal antibody, is known to trigger DCD via LMP in B‐cell malignancies.^2^ However, the cellular factors that regulate this pathway remain poorly defined.^3^ To systematically identify modulators of OBI‐induced cytotoxicity, we conducted a customised CRISPRi screen in Raji B cells targeting 2318 druggable genes^4^ (Figure 1A). Following iterative rounds of OBI treatment and selection, next‐generation sequencing (NGS) analysis revealed genes whose knockdown either sensitised cells to or conferred resistance against OBI‐induced LMP and DCD (Figure 1B). Using Model‐based Analysis of Genome‐wide CRISPR/Cas9 Knockout (MAGeCK, Table S1), we ranked genes based on their depletion or enrichment following OBI treatment, identifying key regulators of LMP and DCD (Figure 1C). Essential genes critical for cell survival were excluded, as their sgRNAs were predominantly depleted. To prioritise clinically relevant targets, we applied a selection strategy: (1) applying FDR cutoff of 25%, (2) excluding essential genes based on the DepMap database and Raji B‐cell essential gene list, specifically selecting genes with DepMap gene essentiality scores < –1 and at least three high‐quality sgRNAs (resulting in 97 genes, Table S2), and (3) focus on druggable targets with available inhibitors. This yielded 15 candidate genes for further validation. The sgRNA enrichment profiles and their relative importance are summarised in a box plot (Figure 1D and E).

**FIGURE 1:**
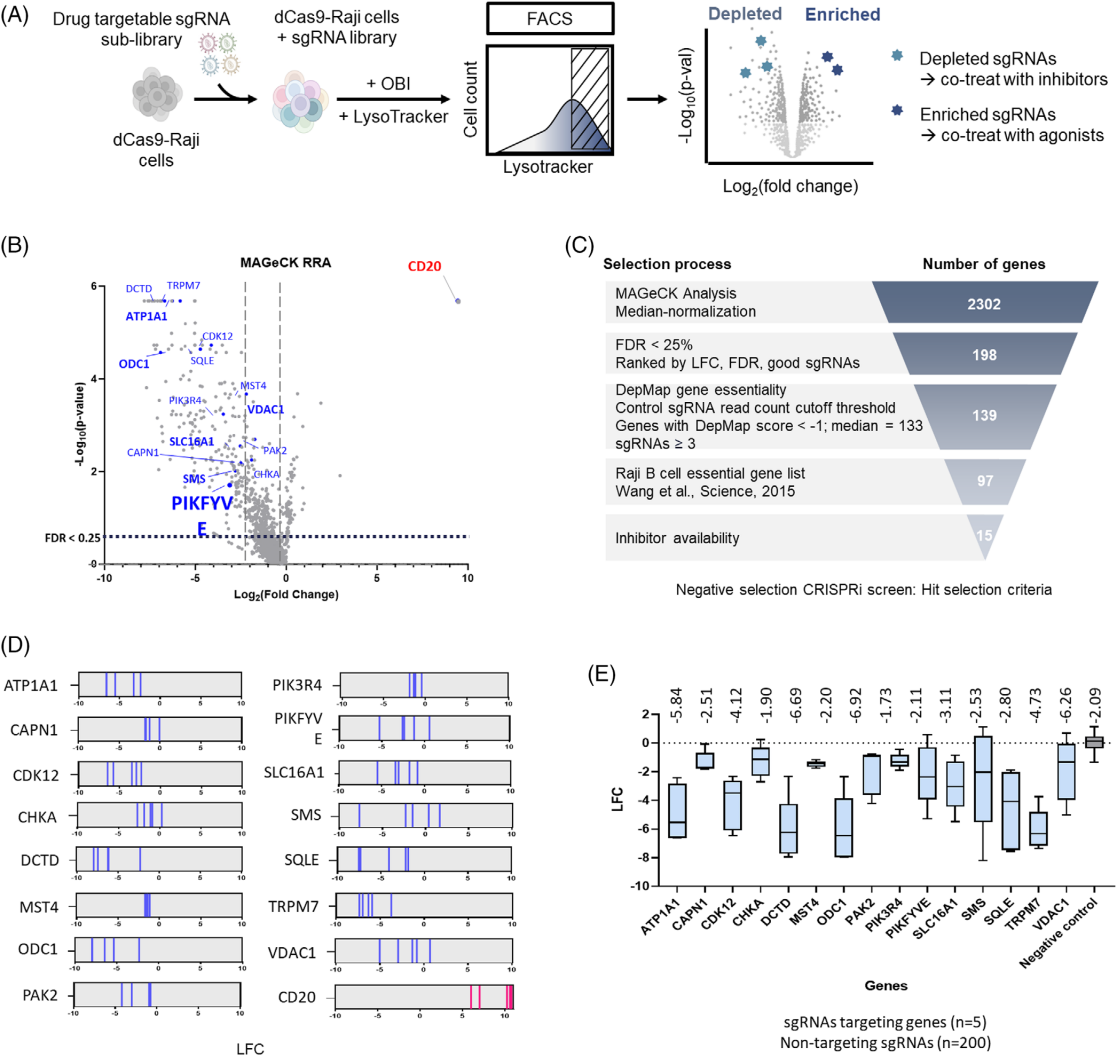
**CRISPRi reveals modulators of OBI‐induced LMP. (A)** Overview of CRISPRi screening. A sub‐library of sgRNAs targeting kinases, phosphatases, and drug target genes was delivered into dCas9‐Raji cells via a lentiviral system to create an sgRNA library pool. dCas9‐library‐Raji cells were treated with 10 µg/mL OBI for 4 h, stained with 50 nM Lysotracker (LT) for 30 min, and LT‐positive cells were sorted using FACS Aria III. Selected cells were expanded and re‐sorted through the same procedure for a total of 5 cycles, with the sgRNA sequences of the final selected cells analysed by NGS. **(B)** Volcano plot illustrating gene‐level enrichment and depletion following CRISPRi screening. Data were analysed using the MAGeCK algorithm to determine the statistical significance (–log₁₀*p*‐value) and magnitude (log₂ fold‐change) of sgRNA abundance in the selected cell population. Significantly enriched genes, indicative of a positive selection phenotype, are highlighted in red, whereas significantly depleted genes, corresponding to negative selection, are shown in blue. Labelled genes represent top candidates based on statistical ranking. **(C)** Stepwise selection of genes depleted by OBI‐induced lysosomal membrane permeabilisation (LMP). MAGeCK analysis identified 2302 candidate genes following median normalisation. Genes with FDR < 25% were further ranked by LFC, FDR, and sgRNA quality (*n* = 198). Essential genes were filtered using DepMap data (DepMap score < –1; higher than 3 sgRNAs), yielding 139 candidates. Cross‐referencing with the Raji B‐cell essential gene list refined the set to 97 genes. Final selection of 15 genes was based on inhibitor availability. **(D)** sgRNA enrichment plot displaying relative changes of 5 sgRNAs per gene. Depleted genes are indicated in blue, and enriched genes such as CD20 are shown in pink. **(E)** Box plot representing average log fold‐change (LFC) per gene. Numbers above indicate the median LFC for each gene.

To validate this finding, we generated single‐knockdown Raji cell lines for 14 candidate genes and confirmed their knockdown efficiency (Figure 2A, Tables S3 and S4; CDK12 was excluded due to the inability to establish a stable knockdown line). Among these, six genes (DCTD, SMS, ATP1A1, SLC16A1, ODC1, and PIKfyve) significantly increased LMP and DCD, reinforcing their potential as modulators of lysosomal disruption (Figure 2B–D). Apilimod, a selective PIKfyve inhibitor, showed the strongest enhancement of OBI‐induced DCD with minimal cytotoxicity alone (Figure 2E and F). Additionally, Apilimod dose‐dependently enhanced DCD induced by OBI and RTX, indicating that PIKfyve inhibition broadly amplifies anti‐CD20‐mediated cytotoxicity (Figure 2G–J). Further, Umbralisib (a PI3Kδ inhibitor) enhanced OBI‐induced cytotoxicity, whereas Acalabrutinib (a BTK inhibitor) had no effect, suggesting that the PI3K pathway, including PIKfyve, contributes to OBI efficacy (Figure S4).

**FIGURE 2:**
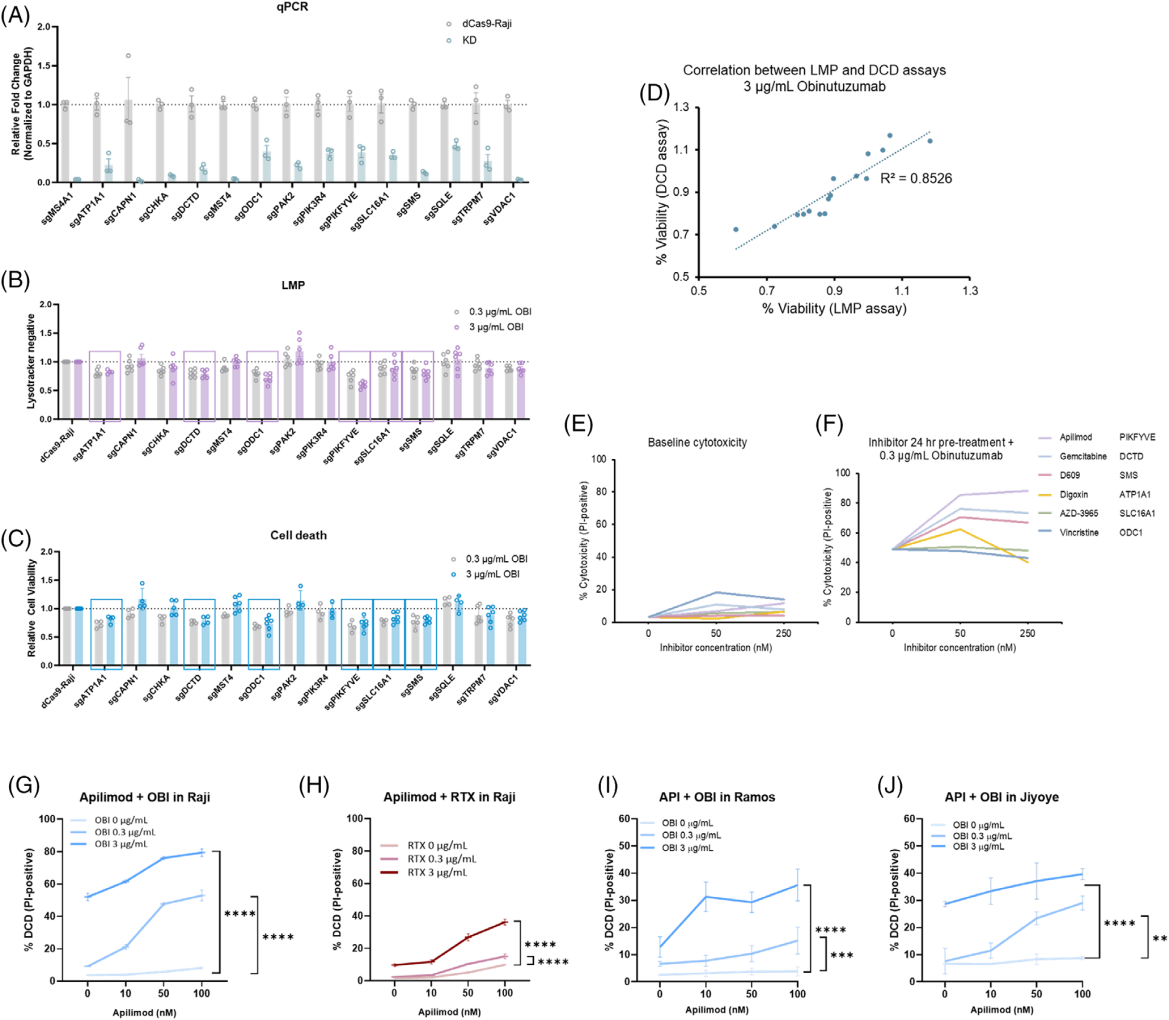
**Inhibition of PIKfyve activity is a synergistic target for amplifying OBI‐induced LMP. (A)** Validation of mRNA expression reduction in dCas9‐Raji‐sgRNA cell lines stably expressing sgRNAs for 14 genes selected screening, compared to gene expression levels in dCas9‐Raji cells, as determined by quantitative PCR (qPCR). **(B, C)** LMP and DCD analysis in single sgRNA stably expressing dCas9‐Raji‐sgRNA cell lines. Six genes were identified that either showed consistent or dose‐dependent increases in LMP or DCD compared to cell survival rates in dCas9‐Raji cells treated with.3 µg/mL and 3 µg/mL OBI. **(D)** Positive correlation between LMP and DCD ratios in the context of 3 µg/mL OBI treatment. **(E, F)** Degree of DCD as determined by Propidium Iodide (PI) positive cell count analysis with 50 nM or 250 nM of inhibitors for the six selected genes, applied singly (E) or in combination with OBI (F). For single treatments, each inhibitor was pre‐treated for 24 h (E), and for combination with OBI treatments (F), cells were pre‐treated with each inhibitor for 24 h followed by treatment with.3 µg/mL OBI to measure cell death rates. **(G, H)** Increase in DCD by the combination treatment of Apilimod with OBI or RTX. The degree of DCD by dose‐dependent pre‐treatment of Apilimod (0, 10, 50, and 100 nM) for 24 h followed by dose‐dependent treatment of OBI (G), or RTX (H) (0,.3 µg/mL, and 3 µg/mL) for 4 h. **(I, J)** Increase in DCD by the combination treatment of Apilimod with OBI in two different B‐cell lymphoma cell lines. The degree of DCD in Ramos (I) and Jiyoye (J) cells by dose‐dependent pre‐treatment of Apilimod (0, 10, 50, and 100 nM) for 24 h followed by dose‐dependent treatment of OBI (0,.3 µg/mL, and 3 µg/mL) for 4 h. Statistical significance was calculated for the no treatment group and each antibody concentration treatment group at 100 nM of Apilimod condition. Data are presented as mean ± SEM, and calculated *p*‐values are shown for the difference between condition treated with 100 nM Apilimod without antibodies and with each concentration of antibody.

Given that PIKfyve regulates lysosomal membrane integrity,^5^ we investigated whether its inhibition affects lysosomal function and DCD. Apilimod, disrupts lysosomal fission and induces persistent lysosomal hypertrophy.^5^ Notably, the extend of OBI‐induced cell death amplified by Apilimod was disproportionately greater than the increase in LMP. This pattern contrasts with the response to L‐Leucyl‐L‐Leucine methyl ester (LLOMe), where high concentrations induced both LMP and DCD, while low concentrations triggered substantial LMP with minimal DCD (Figure 3A), likely due to activation of lysosomal membrane repair mechanisms.^6^ These findings suggest that OBI‐induced LMP – particularly when enhanced by Apilimod – may exceed the threshold of damage that is reparable, resulting in irreversible lysosome‐dependent cell death. The synergistic increase in OBI‐induced LMP and DCD by Apilimod was consistently observed across multiple B‐cell lymphoma cell lines, including Raji, Ramos, and Jiyoye (Figure 3A–C). To further validate LMP as the underlying mechanism, we assessed cathepsin B release as a marker of lysosomal disruption. We observed a marked increase in cytosolic cathepsin signal in cells co‐treated with OBI and Apilimod compared to OBI alone (Figure 3D and E), providing direct evidence of LMP‐associated protease release.

**FIGURE 3:**
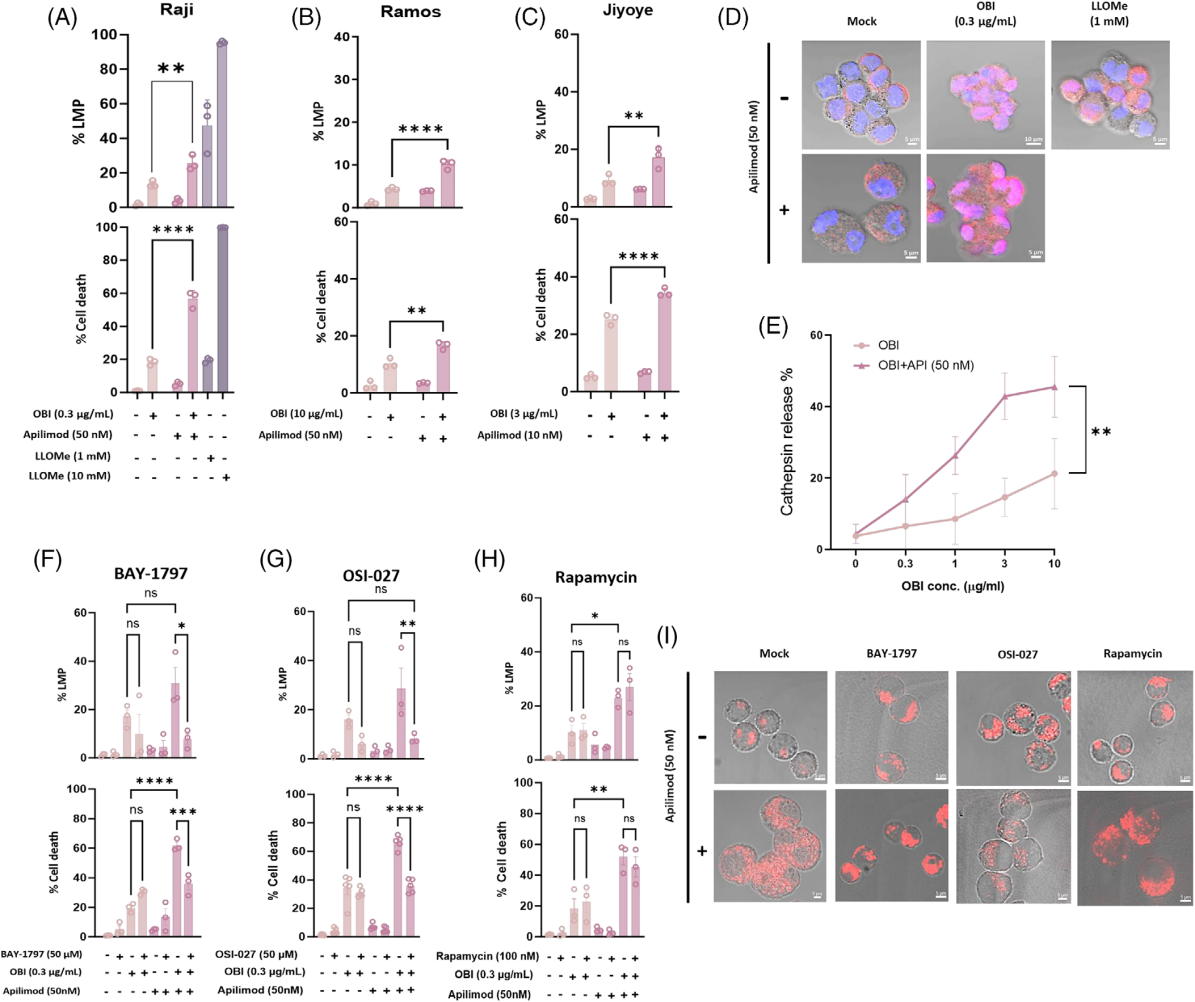
**OBI and Apilimod induce LMP‐mediated cell death, validated across multiple cell lines and by cathepsin release. (A)** The combinational effects of obinutuzumab (OBI,.3 µg/mL) and Apilimod (50 nM) significantly enhanced direct cell death (DCD) and lysosomal membrane permeabilisation (LMP) in Raji cells. LLOMe (1 and 10 mM) were used as a positive control to induced LMP. **(B, C)** The synergistic effect of Apilimod and OBI on DCD and LMP was confirmed in two additional B‐cell lymphoma cell lines, Ramos (B) and Jiyoye (C). **(D)** Immunofluorescence analysis of cathepsin B localisation revealed increased cytosolic release upon OBI + Apilimod co‐treatment, compared to OBI alone. LLOMe (1 mM) served as a reference for partial lysosomal disruption. **(E)** Apilimod markedly enhances cathepsin B release across all tested doses of OBI, indicating amplification of LMP. **(F–H)** The impact of P2 × 4 receptor inhibition (BAY‐1797, 50 µM), dual mTORC1/2 inhibition (OSI‐027, 50 µM), and selective mTORC1 inhibition (rapamycin, 100 nM) was assessed on DCD and LMP in the context of OBI + Apilimod treatment. **(I)** Lysosomal morphology and LysoTracker fluorescence intensity were assessed by confocal microscopy following LysoTracker Deep Red staining. All drugs were incubated overnight. Scale bars, 5 µm. Data are presented as mean ± SEM, and *p*‐values are compared between the OBI alone and OBI, API combinational treatment group and each inhibitor co‐treatment group. A *p*‐value < .05 is considered statistically significant. ‘ns’ indicates no significance.

To explore the mechanisms underlying this effect, we assessed lysosomal trafficking and signalling pathways. BAY‐1797, a P2X4 receptor inhibitor, significantly reduced Apilimod‐enhanced DCD, suggesting that endosomal‐lysosomal trafficking is essential for this process (Figure 3F). Furthermore, OSI‐027, a dual mTORC1/2 inhibitor,^7^ completely abrogated Apilimod's effect, whereas Rapamycin had no significant impact (Figure 3G and H), our results suggest that mTORC2, rather than mTORC1, may be involved in the regulation of lysosomal homeostasis during cytotoxic stress caused by OBI. Confocal imaging confirmed that Apilimod‐induced lysosomal enlargement was evident in all conditions except BAY‐1797 treatment, and only OSI‐027 reduced lysosome intensity in imaging (Figure 3I). These findings indicate that PIKfyve inhibition by Apilimod perturbs lysosomal homeostasis, and suggest that mTOR2 signalling and lysosomal trafficking may contribute to regulating OBI‐induced cytotoxicity.

Our study identifies PIKfyve as a critical regulator of OBI‐induced LMP and DCD, providing mechanistic insights into how lysosomal integrity governs B‐cell lymphoma sensitivity to anti‐CD20 therapy. The discovery that PIKfyve inhibition potentiates OBI cytotoxicity highlights its potential as a co‐therapeutic target for lymphoma treatment. Notably, Apilimod not only enhanced OBI‐induced DCD but also potentiated RTX‐induced cytotoxicity, suggesting a broader role for PIKfyve inhibition in anti‐CD20 monoclonal antibody therapy. The mechanistic link between PIKfyve inhibition and lysosomal dysfunction^7^ suggests that disrupting lysosomal integrity may be a novel strategy to enhance lymphoma therapy. Currently, PIKfyve inhibitors such as Apilimod are in clinical trials for B‐cell malignancies.^8^ The results of this study may support further preclinical and clinical evaluation of Apilimod in combination with anti‐CD20 therapies.

More precise genetic and pharmacologic analyses are needed to dissect the exact contribution of each mTOR complex to the increased OBI‐induced cell death. However, the increase in OBI‐induced cell death by Apilimod, which is not inhibited by rapamycin but is inhibited by OSI‐027 in this study, suggests that mTORC2 rather than mTORC1 may be involved in the regulation of lysosomal homeostasis during LMP stress. However, the increase in OBI‐induced apoptosis by apilimod, which is not inhibited by rapamycin but is inhibited by OSI‐027, in this study suggests that mTORC2 rather than mTORC1 may be involved in the regulation of lysosomal homeostasis during LMP stress in this process. Recent studies have shown that mTORC2 prevents membrane stress‐induced apoptosis by regulating lipid composition and membrane tension.^9^ Given that PIKfyve inhibition causes lysosomal swelling and destabilisation,^10^ the fact that OSI‐027 inhibits OBI cell death enhanced by apilimod suggests that mTORC2 may play a potential role in maintaining lysosomal integrity under stress conditions.

Future studies should explore whether PIKfyve inhibition enhances anti‐CD20 efficacy across diverse B‐cell lymphoma subtypes, including diffuse large B‐cell lymphoma (DLBCL). Additionally, the long‐term impact of lysosomal destabilisation on lymphoma progression and immune responses warrants further investigation.

## CONCLUSION

Our study establishes PIKfyve inhibition as a potent enhancer of OBI‐induced DCD, uncovering lysosomal dynamics and potential involvement of mTORC2 in this process. These findings provide a strong mechanistic basis for rational combination therapies in B‐cell malignancies, with implications for improving antibody‐based cancer treatments

## AUTHOR CONTRIBUTIONS

YK performed and analysed most of the iCas9 screening experiments, which reveal Apilimod as an OBI combination drug. JO performed both LMP and DCD‐related experiments with various lysosome‐regulating drugs. JRK helped the experiment using CLL cell from patient. JYK organised and supervised the whole project. YK helped and reproduced Lysotracker assays, DL supervised sgRNA library screening strategy as well as many technical processes. YK, DL, JO and JYK wrote the manuscript.

## CONFLICT OF INTEREST STATEMENT

All authors have declared that no competing interest exists.

## FUNDING

This work was supported by grants from the National Research Foundation of Korea, Project Nos. NRF‐2019R1A2C1086348 and RS‐2023‐00220853 to J.Y.K. This work was also supported by a faculty research grant from the Yonsei University College of Medicine (6‐2022‐0044) for J. Y K. The funders had no role in study design, data collection and analysis, decision to publish, or preparation of the manuscript.

## ETHICS STATEMENT

The study protocol involving human samples was approved by the Yonsei University Institutional Review Board (IRB No. 4‐2022‐0471), and informed consent was obtained from all participants in accordance with the Declaration of Helsinki.

## CONSENT TO PARTICIPATE

Patient‐derived PBMC samples were isolated with approval of Yonsei University Institutional Review Committee after obtaining informed consent, under IRP procedure (#4‐2022‐0471).

## Supporting information



Supporting Information

Supporting Information

Supporting Information
