# Supplementary material for: CRISPRi screening identifies PIKfyve as a co‐therapeutic target for obinutuzumab
Source: Clin Transl Med. 2025 May 7;15(5):e70333. doi: 10.1002/ctm2.70333 (PMC12059204; doi:10.1002/ctm2.70333)
Supplement: Supplementary file 1 — Supporting Information [file CTM2-15-e70333-s001.pdf]

Supplementary Table 1. CRISPRi screen results analyzed by MAGeCK algorithm

Label	Reads	Mapped	Mapped %	Total sgRNAs	Zero counts	Gini Index
Control.fastq	11095601	4856811	43.8	13008	33	0.0695
Sample.fastq	7908527	3457636	43.7	13008	463	0.1596

Supplementary Table 2. List of all the 97 sgRNA target genes

Gene	Name	LFC	FDR	P-value
AARS2	Alanyl-TRNA Synthetase 2, Mitochondrial	-4.159	0.097372	5.53E-03
ACACA	Acetyl-CoA Carboxylase Alpha	-4.174	0.050080	2.34E-03
AHCY	Adenosylhomocysteinase	-2.902	0.014999	4.20E-04
ARF1	ADP Ribosylation Factor 1	-2.879	0.134971	8.95E-03
ARF6	ADP Ribosylation Factor 6	-2.575	0.010162	2.38E-04
ARL1	ADP Ribosylation Factor Like GTPase 1	-7.382	0.000309	2.07E-06
ARPC5	Actin Related Protein 2/3 Complex Subunit 5	-1.786	0.060674	3.07E-03
ATP1A1	ATPase Na+/K+ Transporting Subunit Alpha 1	-5.835	0.000309	2.07E-06
ATP5B	ATP synthase F1 subunit beta	-2.952	0.018519	6.27E-04
B4GALT1	Beta-1,4-galactosyltransferase 1	-2.415	0.117346	7.33E-03
BUB1	BUB1 Mitotic Checkpoint Serine/Threonine Kinase B	-6.247	0.016708	4.94E-04
CAPN1	Calpain 1	-2.507	0.006582	6.58E-03
CDK12	Cyclin dependent kinase 12	-4.123	0.001591	1.86E-05
CHKA	Choline kinase alpha	-1.904	0.098113	5.64E-03
CIB3	Calcium And Integrin Binding Family Member 3	-2.115	0.117346	7.44E-03
CLPB	Caseinolytic Mitochondrial Matrix Peptidase Chaperone Subunit B	-7.689	0.007280	1.55E-04
CMAS	Cytidine Monophosphate N-Acetylneuraminic Acid Synthetase	-4.777	0.032822	1.37E-03
COX5A	Cytochrome C Oxidase Subunit 5A	-3.862	0.069814	3.70E-03
COX8A	Cytochrome C Oxidase Subunit 8A	-2.184	0.195116	1.46E-02
CS	Citrate Synthase	-1.921	0.085796	4.66E-03
DCPS	Decapping Enzyme, Scavenger	-5.799	0.018088	5.89E-04
DCTD	DCMP Deaminase	-6.690	0.000309	2.07E-06
DUSP12	Dual Specificity Phosphatase 12	-2.889	0.062799	3.25E-03
ESD	S-formylglutathione hydrolase	-4.623	0.001591	1.86E-05
ETNK1	Ethanolamine Kinase 1	-2.665	0.259739	2.16E-02
FECH	Ferrochelatase	-4.468	0.011680	3.12E-04
FGFR1	Fibroblast growth factor receptor 1	-1.489	0.162605	1.15E-02
G6PC3	Glucose-6-Phosphatase Catalytic Subunit 3	-2.354	0.042032	1.83E-03
GABRR2	Gamma-Aminobutyric Acid Type A Receptor Subunit Rho2	-1.824	0.045202	2.02E-03
GALE	UDP-galactose-4-epimerase	-2.220	0.183590	1.35E-02
GCSH	Glycine Cleavage System Protein H	-2.299	0.022363	8.13E-04
GFER	Growth Factor, Augmenter Of Liver Regeneration	-6.313	0.001303	1.03E-05
GIT2	GIT ArfGAP 2	-2.045	0.203218	1.53E-02
GLS	Glutaminase	-3.560	0.006291	1.26E-04
GNE	Glucosamine (UDP-N-Acetyl)-2-Epimerase/N-Acetylmannosamine Kinase	-3.544	0.081395	4.39E-03
GSS	Glutathione Synthetase	-4.538	0.011605	2.96E-04
HK2	Hexokinase 2	-7.602	0.000309	2.07E-06
INPP5D	Inositol Polyphosphate-5-Phosphatase D	-3.466	0.018088	5.77E-04
IP6K1	Inositol Hexakisphosphate Kinase 1	-2.186	0.187590	1.39E-02
KIDINS220	Kinase D interacting substrate 220	-5.013	0.000874	6.20E-06
LARS2	Leucyl-TRNA Synthetase 2, Mitochondrial	-5.769	0.001602	2.27E-05
LSS	Lanosterol Synthase	-5.162	0.024483	9.41E-04
MAP3K14	Mitogen-Activated Protein Kinase Kinase Kinase 14	-2.468	0.001650	2.69E-05
MST4	Mammalian STE20-like protein kinase 4	-2.196	0.009443	2.13E-04
MTFMT	Mitochondrial Methionyl-TRNA Formyltransferase	-3.928	0.030057	1.23E-03
MTRR	Methionine synthase reductase	-1.814	0.011680	3.12E-04
NARS2	Asparaginyl-TRNA Synthetase, Cytoplasmic	-6.286	0.001591	1.86E-05
NDUFA1	NADH:Ubiquinone Oxidoreductase Subunit A1	-3.706	0.064911	3.39E-03
NDUFA5	NADH:Ubiquinone Oxidoreductase Subunit A5	-6.786	0.048390	2.24E-03
NDUFA8	NADH:Ubiquinone Oxidoreductase Subunit A8	-6.939	0.000309	2.07E-06
NDUFA9	NADH:Ubiquinone Oxidoreductase Subunit A9	-3.872	0.010162	2.42E-04
NDUFB10	NADH:Ubiquinone Oxidoreductase Subunit B10	-3.809	0.130966	8.53E-03
NDUFB4	NADH dehydrogenase 1 beta subcomplex, 4	-3.865	0.001650	2.69E-05
NDUFB6	NADH:Ubiquinone Oxidoreductase Subunit B6	-4.077	0.204447	1.55E-02
NDUFC2	NADH:Ubiquinone Oxidoreductase Subunit C2	-2.377	0.187028	1.38E-02
NDUFS2	NADH:Ubiquinone Oxidoreductase Core Subunit S2	-5.188	0.091436	5.12E-03
NDUFS5	NADH:Ubiquinone Oxidoreductase Subunit S5	-5.013	0.026764	1.07E-03
NDUFS8	NADH:Ubiquinone Oxidoreductase Core Subunit S8	-4.759	0.057485	2.79E-03
NME6	NME/NM23 Nucleoside Diphosphate Kinase 6	-2.679	0.141211	9.56E-03
NMNAT1	Nicotinamide Nucleotide Adenylyltransferase 1	-7.123	0.001591	1.86E-05
ODC1	Ornithine Decarboxylase 1	-6.918	0.001650	2.69E-05
PAK2	P21 (RAC1) Activated Kinase 2	-1.725	0.045202	2.02E-03
PDHA1	Pyruvate Dehydrogenase E1 Subunit Alpha 1	-7.083	0.000309	2.07E-06
PDHB	Pyruvate Dehydrogenase E1 Subunit Beta	-5.343	0.005872	1.05E-04
PGK1	Phosphoglycerate Kinase 1	-5.044	0.000309	2.07E-06
PIK3R4	Phosphoinositide-3-Kinase Regulatory Subunit 4	-2.106	0.105761	6.32E-03
PIKFYVE	Phosphoinositide Kinase, FYVE-Type Zinc Finger Containing	-3.111	0.240377	1.97E-02
PIP5K1A	Phosphatidylinositol-4-Phosphate 5-Kinase Type 1 Alpha	-2.940	0.006291	1.26E-04
PLK4	Polo like kinase 4	-6.390	0.096920	5.47E-03
PNPO	Pyridoxamine 5'-phosphate oxidase	-3.079	0.009451	2.17E-04
PPP1R15B	Protein Phosphatase 1 Regulatory Subunit 15B	-1.725	0.222693	1.74E-02
PPP1R21	Protein Phosphatase 1 Regulatory Subunit 21	-3.161	0.010488	2.58E-04
PPP1R8	Protein Phosphatase 1 Regulatory Subunit 8	-3.301	0.001650	2.69E-05
PRKRIR	THAP Domain Containing 12	-2.780	0.240377	1.97E-02
PRPF4B	Pre-MRNA Processing Factor 4B	-3.340	0.023121	8.50E-04
PTDSS1	Phosphatidylserine synthase 1	-5.033	0.001591	1.86E-05
PTPLB	Very-long-chain (3R)-3-hydroxyacyl-CoA dehydratase 2 (HACD2)	-5.019	0.000309	2.07E-06
PTPN6	Protein Tyrosine Phosphatase Non-Receptor Type 6	-3.235	0.086521	4.77E-03
RALA	RAS Like Proto-Oncogene A	-2.507	0.183590	1.35E-02
RPS6KB1	Ribosomal protein S6 kinase beta-1	-1.777	0.134971	8.94E-03
SCYL1	SCY1 Like Pseudokinase 1	-2.475	0.247850	2.04E-02
SEPSECS	Sep (O-phosphoserine) tRNA:Sec (selenocysteine) tRNA synthase	-7.540	0.000309	2.07E-06
SLC7A1	Solute Carrier Family 7 Member 1	-2.264	0.018519	6.51E-02
SMS	Spermine synthase	-2.801	0.141844	9.68E-03
SOD1	Superoxide Dismutase 1	-6.478	0.001602	2.27E-05
SQLE	Squalene epoxidase	-4.725	0.001602	2.27E-05
SSB	Small RNA Binding Exonuclease Protection Factor La	-4.782	0.046159	2.08E-03
TK1	Thymidine Kinase 1	-2.161	0.222772	1.76E-02
TP53RK	TP53 regulating kinase	-2.450	0.060113	2.98E-03
TPK1	Thiamin Pyrophosphokinase 1	-7.813	0.000309	2.07E-06
TRAPPC3	Trafficking Protein Particle Complex Subunit 3	-2.634	0.225892	1.81E-02
TRIM33	Tripartite Motif Containing 33	-2.216	0.042103	1.85E-03
TRPM7	Transient Receptor Potential Cation Channel Subfamily M Member 7	-6.264	0.000309	2.07E-06
UQCRQ	Ubiquinol-Cytochrome C Reductase Complex III Subunit VII	-1.749	0.098126	5.70E-03
VDAC1	Voltage dependent anion channel 1	-2.091	0.152805	1.05E-02
VRK1	VRK Serine/Threonine Kinase 1	-3.084	0.040517	1.74E-03
WARS2	Tryptophanyl TRNA Synthetase 2, Mitochondrial	-4.139	0.089742	4.99E-03

Supplementary Table 3. List of sgRNA oligonucleotide sequences for single-knockdown cell lines

Gene	Direction	Sequence
ATP1A1-4	F	TTG GGAGGGAGCGCAGTAACGGG G TTT AAG AGC
	R	TTA GCT CTT AAAC CCCGTTACTGCGCTCCCTCC CAA CAA G
CAPN1-1	F	TTG GCACCGGGAAGCCAGCCTCA G TTT AAG AGC
	R	TTA GCT CTT AAAC TGAGGCTGGCTTCCCGGTGC CAA CAA G
CHKA	F	TTG GCGGGCGGCCGCAGCGCGAG G TTT AAG AGC
	R	TTA GCT CTT AAAC CTCGCGCTGCGGCCGCCCGC CAA CAA G
DCTD-3	F	TTG GGCGCGGAGCCGGCACCGGA G TTT AAG AGC
	R	TTA GCT CTT AAAC TCCGGTGCCGGCTCCGCGCC CAA CAA G
MST4-5	F	TTG GAGGGCCGCCGAACTACCCC G TTT AAG AGC
	R	TTA GCT CTT AAAC GGGGTAGTTCGGCGGCCCTC CAA CAA G
ODC1-5	F	TTG GTAGGGAGCGGCGTGCCGTG G TTT AAG AGC
	R	TTA GCT CTT AAAC CACGGCACGCCGCTCCCTAC CAA CAA G
PAK2-5	F	TTG GCGGAGTCCTGCGCACGCCA G TTT AAG AGC
	R	TTA GCT CTT AAAC TGGCGTGCGCAGGACTCCGC CAA CAA G
PIK3R1-1	F	TTG GCC AGC AGC TGG AGC GGA GT G TTT AAG AGC
	R	TTA GCT CTT AAA CAC TCC GCT CCA GCT GCT GGC CAA CAA G
PIKFYVE	F	TTG GAGTCGGCCCCCGAGAGCGG G TTT AAG AGC
	R	TTA GCT CTT AAAC CCGCTCTCGGGGGCCGACTC CAA CAA G
SLC16A1-4	F	TTG GTGGCTAGCTGCGTGGGTAC G TTT AAG AGC
	R	TTA GCT CTT AAAC GTACCCACGCAGCTAGCCAC CAA CAA G
SMS-2	F	TTG GCTGGGAGTGTGCTGCGCCC G TTT AAG AGC
	R	TTA GCT CTT AAAC GGGCGCAGCACACTCCCAGC CAA CAA G
SQLE-5	F	TTG GCACCAGCATCCCTCGCGGG G TTT AAG AGC
	R	TTA GCT CTT AAAC CCCGCGAGGGATGCTGGTGC CAA CAA G
TRPM7-1	F	TTG GCGGCCTGTAGCCATCTATC G TTT AAG AGC
	R	TTA GCT CTT AAAC GATAGATGGCTACAGGCCGC CAA CAA G
VDAC1-2	F	TTG GCCCGCCGCCACATCCTCTG G TTT AAG AGC
	R	TTA GCT CTT AAAC CAGAGGATGTGGCGGCGGGC CAA CAA G

Supplementary Table 4. qPCR primers used for reduced gene expression in 14 single-knockdown cell lines

No.	Gene		Primer sequences	Length (bp)	Tm (°C)	Amplicon size (bp)	PrimerBank ID
1	ATP1A1	Primer_F	ACAGACTTGAGCCGGGGATTA	21	62.7	100	237681108c1
		Primer_R	TCCATTCAGGAGTAGTGGGAG	21	60		
2	CAPN1	Primer_F	GCCAAGCAGGTGAACTACC	19	60.4	125	311893361c3
		Primer_R	TATGGGTCCACGTTGTTCCAC	21	62		
4	CHKA	Primer_F	ATTACAGGGGATTCGACATTGGA	23	61.1	117	47078275c3
		Primer_R	GCTGTTGTTTCTTGGTGGGAT	21	60.8		
5	DCTD	Primer_F	CCAAATGGGTGCAGTGATGAC	21	61.5	138	61742818c2
		Primer_R	ACTACAGCCTTTCACATCGGT	21	61.1		
6	MST4	Primer_F	ATCTTGTGCAAACCCTGAGTTG	22	61	104	109633024c2
		Primer_R	TTCAATCGCCTGATTCCTGCT	21	61.8		
7	ODC1	Primer_F	TTTACTGCCAAGGACATTCTGG	22	60.2	127	4505488c1
		Primer_R	GGAGAGCTTTTAACCACCTCAG	22	60.3		
8	PAK2	Primer_F	CACCCGCAGTAGTGACAGAG	20	61.9	112	191250770c3
		Primer_R	GGGTCAATTACAGACCGTGTG	21	60.6		
9	PIK3R4	Primer_F	GCTCTTTAGGCAGTATGTGCG	21	61.1	148	116812580c3
		Primer_R	GATGTCCCCATGACGAACTCC	21	62.2		
10	PIKFYVE	Primer_F	ACCTCCGAGCTTGCACATATT	21	61.5	95	295789161c2
		Primer_R	TGAAAGAGCATTCAAGTCTTCCC	23	60.5		
11	SLC16A1	Primer_F	GGTGGAGGTCCTATCAGCAGT	21	62.7	107	115583684c2
		Primer_R	CAGAAAGAAGCTGCAATCAAGC	22	60.4		
12	SMS	Primer_F	TGGGCGGGTGAAACGATTAC	20	62.5	162	311083638c1
		Primer_R	CCAAACTGCTTCGAGTGTAGAA	22	60.2		
13	SQLE	Primer_F	GATGATGCAGCTATTTTCGAGGC	23	60.7	179	62865634c2
		Primer_R	CCTGAGCAAGGATATTCACGACA	23	60		
14	TRPM7	Primer_F	GTTGGAAAGTATGGGGCGGAA	21	62.4	190	296080776c2
		Primer_R	CACACACAACTACTGGAACAGG	22	60.8		
15	VDAC1	Primer_F	CTGACCTTCGATTCATCCTTCTC	21	62.4	75	307133764c1
		Primer_R	CTCCCGCTTGTACCCTGTC	21	63		
