# Supplementary material for: CRISPRi screening identifies PIKfyve as a co‐therapeutic target for obinutuzumab
Source: Clin Transl Med. 2025 May 7;15(5):e70333. doi: 10.1002/ctm2.70333 (PMC12059204; doi:10.1002/ctm2.70333)
Supplement: Supplementary file 2 — Supporting Information [file CTM2-15-e70333-s002.pdf]

Supplementary Figure 1

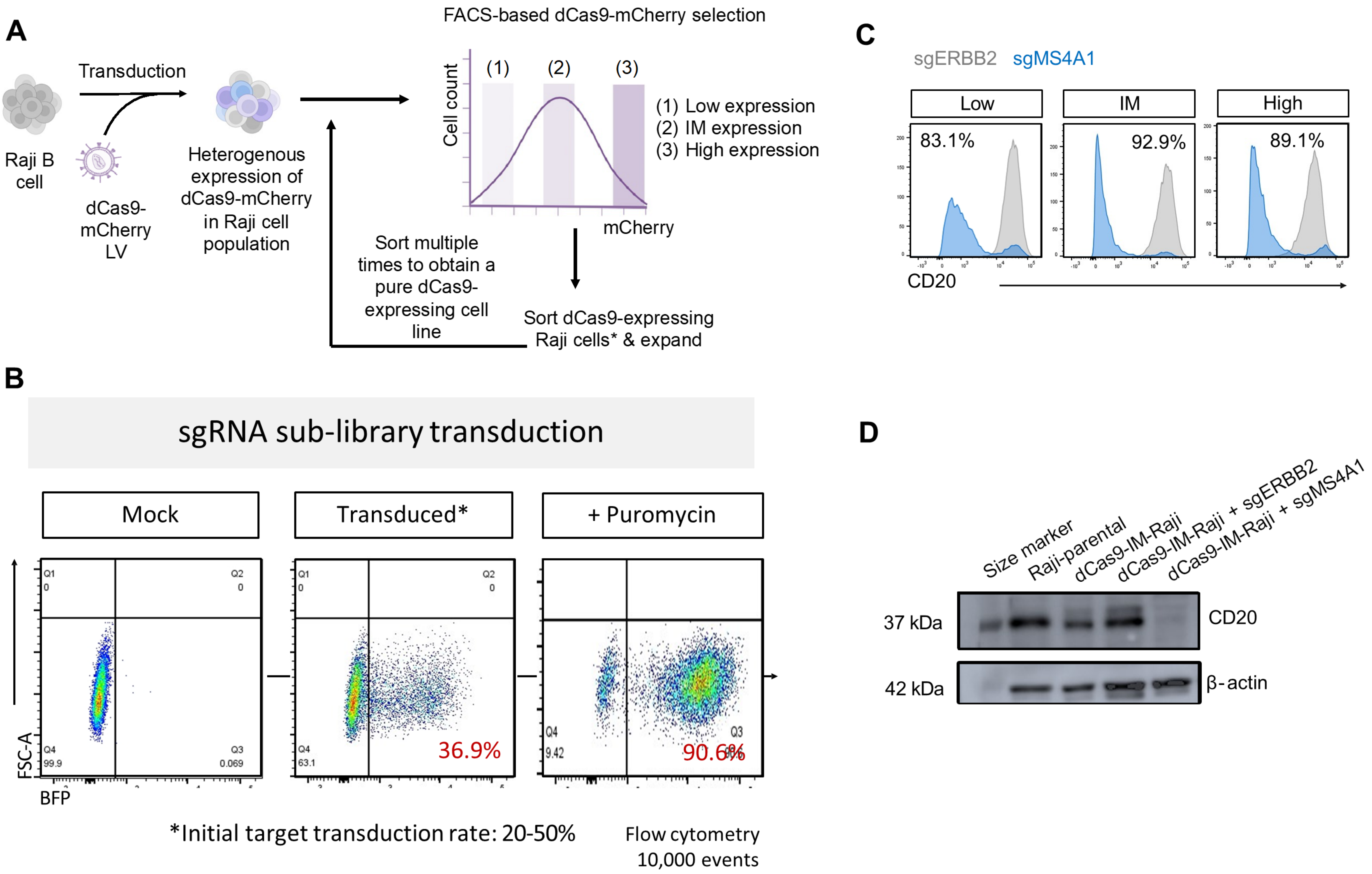

Supplementary Figure 1. Construction of single sgRNA expressing cell library. A. Workflow of dCas9-Raji cell line generation. Raji cells were lentivirally transduced with a dCas9-mCherry Lentivirus, resulting in heterogeneous expression of dCas9-Raji cell line. dCas9-Raji cell line were sorted iterative to obtain high expression populations using FACs. **B.** The library pool construction adapted to have one sgRNA. dCas9 stable-Raji cells were introduced to express the sgRNA library-encoding lentivirus at a transcriptional efficiency of approximately 20-50%. The selection efficiency quantified by flow cytometry after treatment of a low dose of puromycin (0.75 μg/mL puromycin). **C, D.** The specific knockdown efficiency of dCas9-CD20 sgRNA expressed Raji cell via flow cytometry and western blot with anti-CD20 antibody (RTX). β-actin was used for loading control.

Supplementary Figure 2

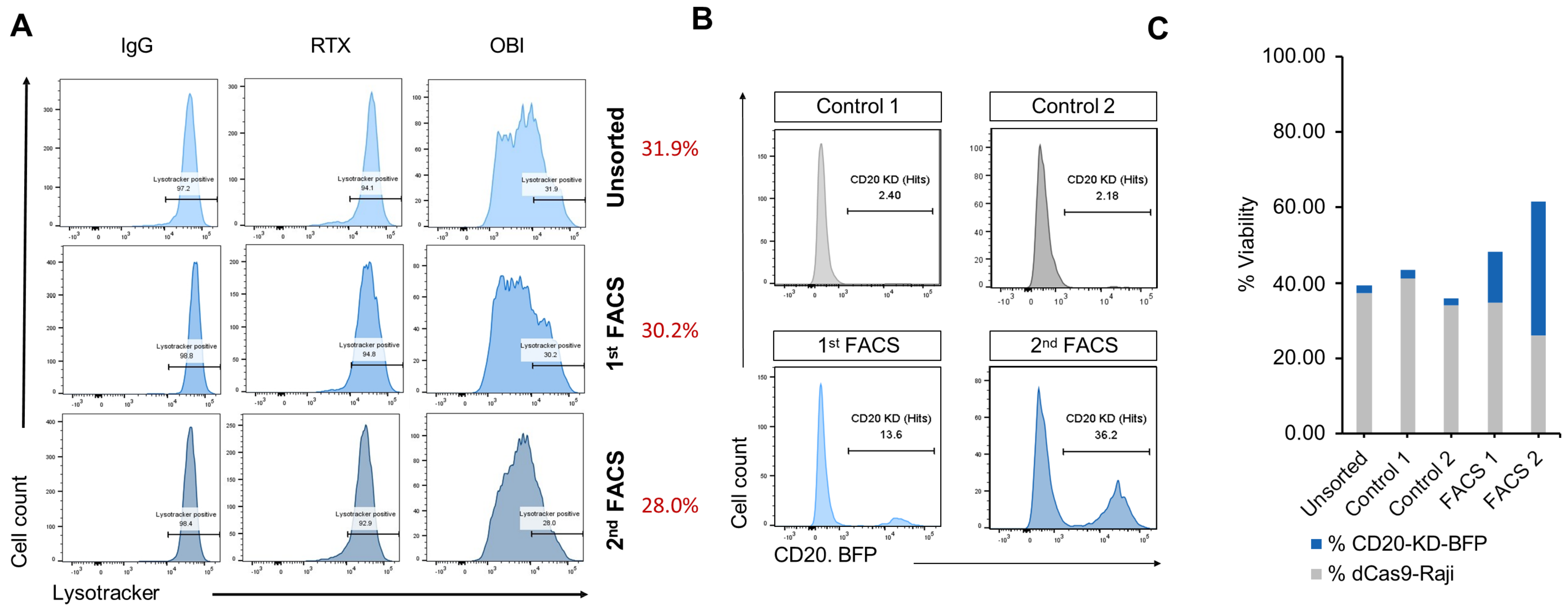

Supplementary Figure 2. Enrichment of specific sgRNA expressing cell resistance to OBI-induced LMP during screening procedure A. Screening workflow was validated via iterative FACS-based selection. FACs-sorted lysotracker-positive dCas9-Raji cells were re-treated with OBI and lysotracker. Lysotracker-negative rates were analyzed by flow cytometry. The 2nd sorted cells were still responsive to obinutuzumab, but the lysotracker histograms shifted more to the right (Lysotracker full positive cell 1st: 30.2%, 2nd : 28.0%) which indicates that cells were beginning resistance. **B.** The increased population of CD20 knockdown (KD) cells (expressing BFP) were shown during the repetitive selections. As selection rounds goes by, the population of BFP-expressing CD20 KD cells were accumulated. Control cells were passed through the FACs sorter to account for FACs-induced cell damage. **C.** Viability of each round cell accompany with percent of CD20 KD cells.

Supplementary Figure 3

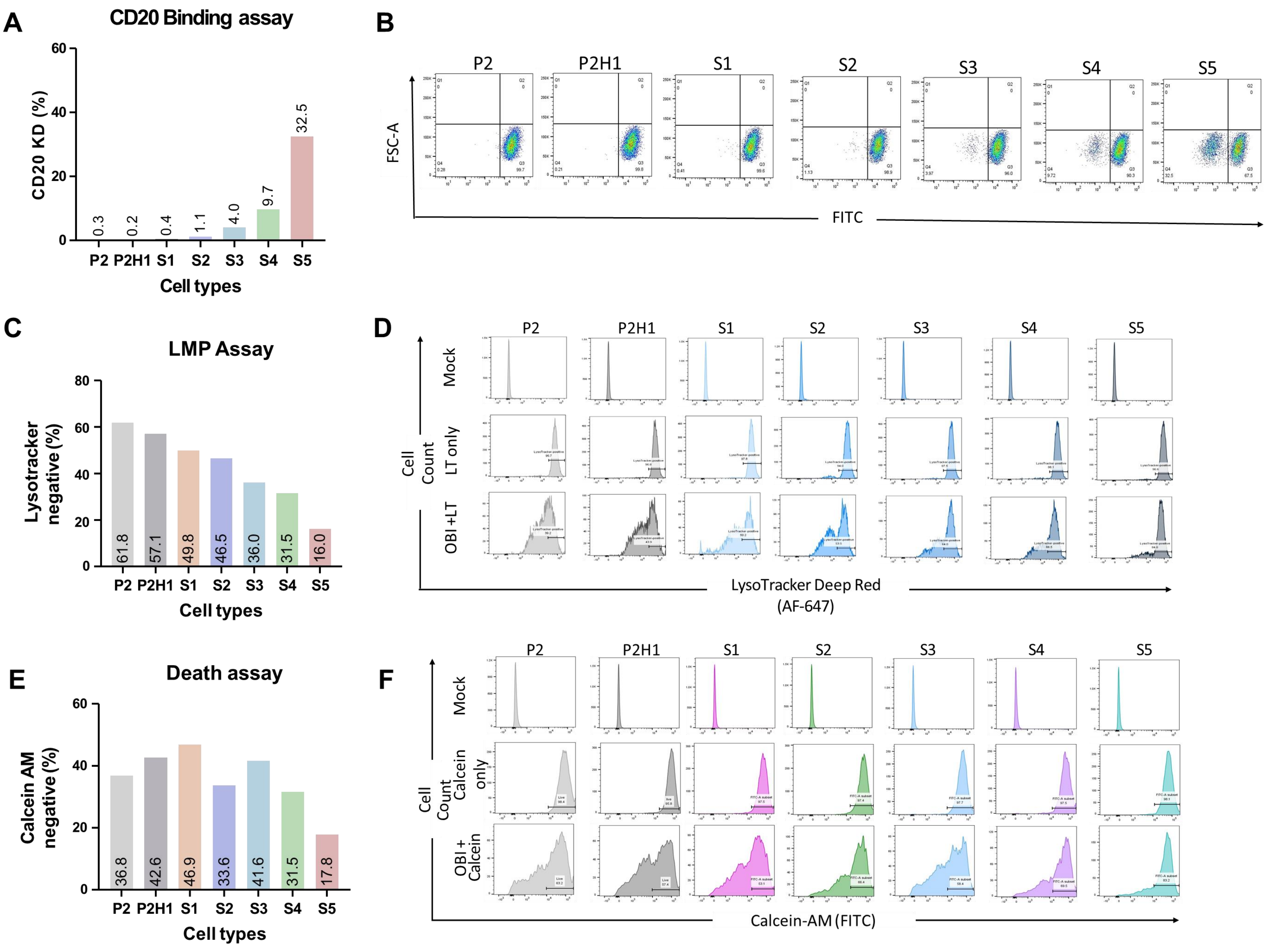

Supplementary Figure 3. Characterization of cells selected through five rounds of screening for resistance to OBI-induced LMP. (A, B) The knockdown of CD20 mRNA expression rate in each round of screening. 2.0 x 10^5 sorted cells were treated with 10 ug/mL RTX and 1:500 goat anti-Human IgG Fc specific FITC-conjugated secondary antibody. (P: dCas9-Raji cell populations. P2H1: dCas9-Raji cell lentivirally transduced with CRISPRi sgRNA library. S: Sorting population of P2H1). C-F. LMP (C, D) and DCD (E, F). 1.0 x 10^5 sorted cells were treated with 10 μg/ml OBI-WT for 4 hr and stained with 50 nM LysoTracker Deep Red (C, D) or calcein-AM (E, F) for 30 min then analyzed by flow cytometry and FlowJo. The representative FACS images were shown in B, D, and F and summarized in A, C, E.

Supplementary Figure 4

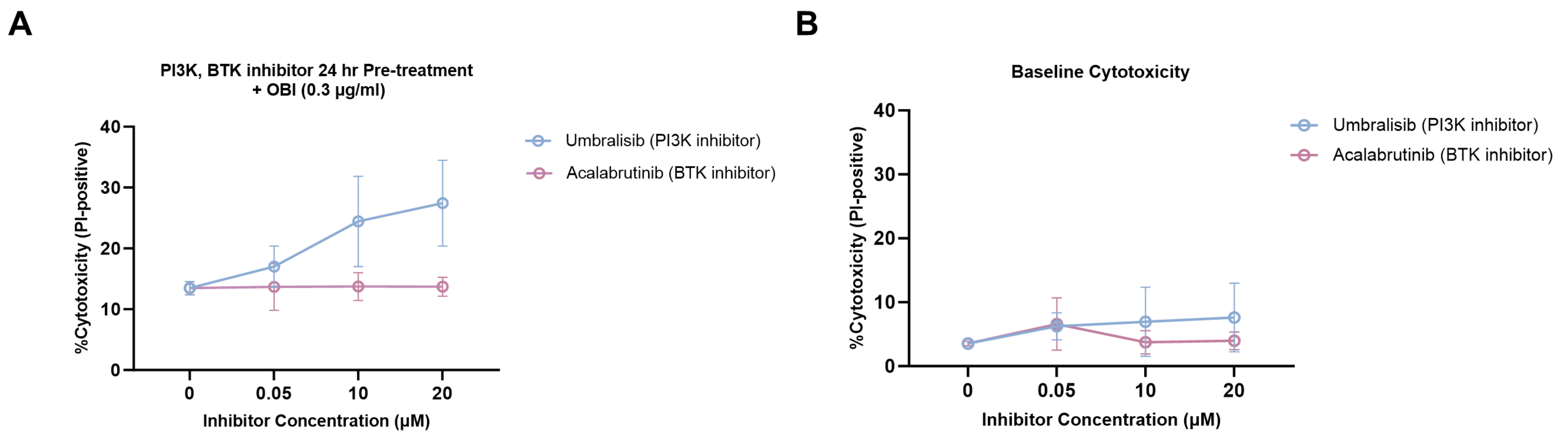

Supplementary Figure 4. Direct cell death (DCD) effects of PI3K and BTK inhibitors in combination with OBI treatment in Raji cells. The combination of Umbralisib with OBI increased DCD, whereas Acalabrutinib did not. (A) For single treatments, each inhibitor was pre-treated for 24 hours, and for combination treatments (B), cells were pre-treated with each inhibitor for 24 hours followed by treatment with 0.3 µg/mL OBI for 4 hours to measure cell death rates. DCD was evaluated through propidium iodide (PI) staining

Supplementary Figure 5

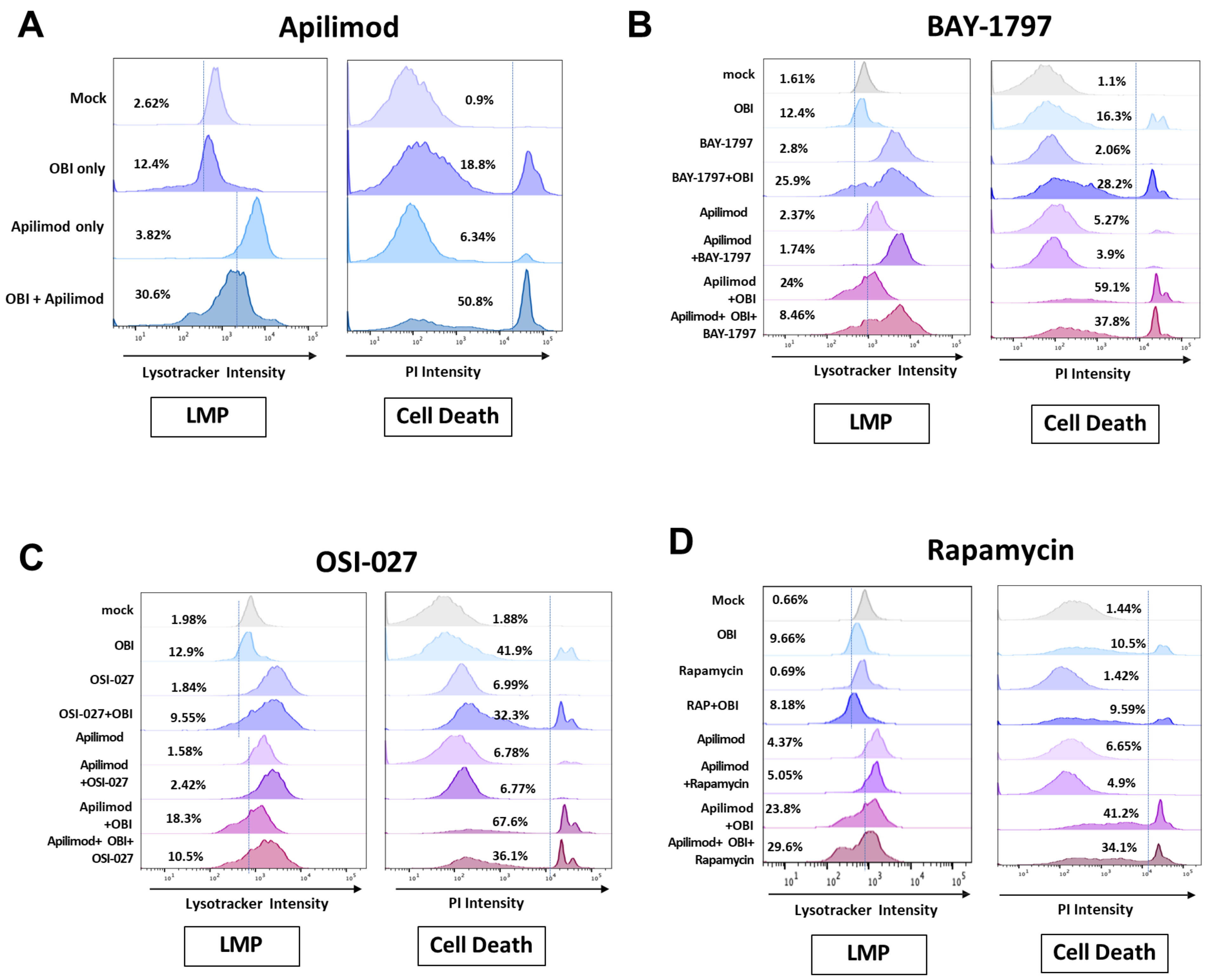

Supplementary Figure 5. FACS histograms illustrating lysosomal membrane permeability (LMP) and cell death (DCD) in Raji cells. Cells were pre-treated with Apilimod and each respective drug, followed by treatment with Obinutuzumab for 4 hours. Lysosomal integrity was assessed using LysoTracker™ Green fluorescence, and direct cell death was evaluated through propidium iodide (PI) staining. The histograms depict fluorescence intensities of Lysotracker and PI under treatment with Apilimod alone **(A)** and in combination with Bay-1797 **(B)**, OSI-027 **(C)**, Rapamycin **(D).**
