# Supplementary material for: CRISPRi screening identifies PIKfyve as a co‐therapeutic target for obinutuzumab
Source: Clin Transl Med. 2025 May 7;15(5):e70333. doi: 10.1002/ctm2.70333 (PMC12059204; doi:10.1002/ctm2.70333)
Supplement: Supplementary file 3 — Supporting Information [file CTM2-15-e70333-s003.docx]

**Materials and methods**

**Cell lines**

Raji B cells (gifted by Prof. Seong Hwan Kim at Chungnam National University), Ramos cells, Jiyoye cells were maintained in Roswell Park Memorial Institute (RPMI)-1640 medium supplemented with 10% fetal bovine serum (FBS) and 1% penicillin-streptomycin. Cells were cultured in a humidified incubator (37 °C, 5% CO_2_). Three cell lines were seeded at a density of 1.0 x 10^5^ cells/mL and passaged every 2-3 days. For cell cultures, RPMI 1640 (Welgene), FBS (Gibco, Life technologies, Carlsbad, CA, USA), and penicillin-streptomycin (Welgene) were used.

**CRISPRi Screening and Validation in Raji B Cells**

To generate the CRISPRi Raji B cell line, Raji B cells were lentivirally transduced with SFFV-dCas9-mCherry-KRAB (Fig. S1A, Addgene #180264) and validated by transducing with either sgMS4A1 or a non-targeting control sgERBB2, followed by selection with 1 μg/mL puromycin (ant-pr-1, Invivogen). A polyclonal dCas9-Raji B cell line was generated by FACS for mcherry-positive cells. Cells were sorted on the BD FACS Aria III cell sorter by mcherry fluorescence intensity, then sorted cells were expanded. Surface CD20 expression was analyzed by flow cytometry to confirm knockdown.

The 50 ng of sgRNA sub-library targeting kinases, phosphatases, and drug targets (a gift from Jonathan Weissman, Addgene #83971) was amplified by SS320 electrocompetent *E.coli* electroporation using the Bio-Rad Gene Pulser II Electroporator (2.4 kV, 25 μF, 300 Ω). The sgRNA-sub library was packaged into lentiviral vectors and subsequently delivered to dCas9-Raji cells via lentiviral transduction. For library construction, 1.3 × 10⁷ dCas9-Raji cells were transduced with an initial target transduction rate of 20-50%, as quantified by flow cytometry for BFP expression, and selected with 0.75 μg/mL puromycin.

For CRISPRi screening, sgRNA-dCas9-Raji cells (maintaining 1,000× library coverage) were treated with 10 μg/mL OBI for 4 hours at 37 °C, stained with 50 nM LysoTracker (LT) Deep Red for 30 minutes at 37°C, and filtered through a 40 μM cell strainer. LT-positive cells were sorted using a BD FACS Aria III, expanded for one week, and subjected to iterative rounds of FACS until most cells were LT-positive. Genomic DNA (gDNA) from control and OBI-treated cells was extracted using Macherey-Nagel’s Nucleospin Blood Midi kit, following the manufacturer’s protocol, and its quality was confirmed via agarose gel electrophoresis. The sgRNA sequences within the gDNA were enriched by a three-step PCR for 23 cycles. PCR products were purified using SPRIselect beads (Beckman and Coulter B23317) and analyzed using an Agilent 4200 TapeStation with the D1000 kit. The first PCR products were sent to Macrogen, an NGS service provider, for adapter ligation using the TruSeq Nano DNA (LMW) library kit and further analyzed on the TapeStation before sequencing. The second PCR products were sequenced on the Illumina NovaSeq platform, where sequences were amplified in situ and read using sequencing by synthesis (SBS) chemistry.

Reads from FASTQ files were trimmed to remove adapter sequences and mapped to the sgRNA library targeting kinases, phosphatases, and drug targets. For both control and OBI-treated samples, a table of sgRNA read counts was generated. Genes were ranked using the Model-based Analysis of Genome-wide CRISPR/Cas9 Knockout (MAGeCK) algorithm. Screen hits were determined based on robust ranking aggregation (RRA) scores, log₂ fold change (LFC), false discovery rate (FDR), DepMap gene perturbation scores, gene expression profiles, and inhibitor availability. Figures were generated using GraphPad Prism v9.

To validate screen hits, individual sgRNAs targeting hit genes were cloned into the CRISPRi/a V2 library parental plasmid (Addgene #84832) and lentivirally transduced into dCas9-Raji B cells. Transduced cells were selected with 1 μg/mL puromycin, and transduction efficiency was assessed by flow cytometry. The resulting individual gene knockdown cell lines were subjected to LMP and direct cell death (DCD) assays, as described.

**CD20 expression level measurement**

2.0×10^5^ Raji cells were resuspended in 100 μL PBS and treated with 10 μg/mL RTX for 30 min at 4 °C. Cells were washed once with PBS. Cells were resuspended with 100 μL PBS treated with 1:500 anti-human IgG Fc**γ**-specific FITC-conjugated secondary antibody (109-095-008, 1:200 dilutions; Jackson Laboratories) and incubated for 30 min at 4 °C. Cells were washed once with PBS and resuspended in PBS. 10,000 events were analyzed by flow cytometry for changes in CD20 content.

**RNA extraction and quantitative PCR (qPCR)**

Raji cells were prepared for RNA extraction using the TRIzol protocol. 3x10^6^ cells were resuspended in 500ul TRIzol and homogenized with vigorous pipetting and vortex. 100ul chloroform was added and the two layers were vortexed to appear clear layers. The aqueous layer was carefully transferred to a new tube and the RNA was washed with ethanol, dried, and eluted in distilled water. The concentration and purity of the RNA samples were checked by nanodrop. cDNA was synthesized using the RNA template, oligo dT primer, and reverse transcriptase. qPCR primers were generated by PrimerBank (Table 4). qPCR was done following standard protocol for AccuPower® 2X GreenStar™ qPCR Master Mix (2405G, BioNEER).

**OBI-drug combinatorial cytotoxicity and Lysosomal membrane permeabilization assays**

5.0×10^4^ cells were seeded in a 96-well plate and pre-treated with the indicated concentrations of the drugs for 24 hours in a humidified incubator (37 °C, 5% CO_2_). Then, the cells were treated with the indicated concentrations of OBI for 4 hours at 37 °C. In parallel, LLOMe was applied at concentrations of 1mM and 10mM for 1 hour under the same conditions. Following treatment, cells were stained with propidium iodide (PI) and LT green DND-26 for 30 min. 10,000 events were analyzed by flow cytometry on BD FACSLyric for changes in the cell morphology. Briefly, cells were first gated for singlets (based on FSC-A vs FSC-H scatter), followed by lymphocytes (based on FSC-A and SSC-A scatter). Cytotoxicity was quantified as the percentage of PI-positive cells. LMP profiles were also analyzed by percentage of lysosome rupture rate occurring under mock and Apilimod conditions.

**Cathepsin B release detection**

To assess the release of cathepsin B, pre-treated cells were harvested and subsequently fixed using BD Cytofix/Cytoperm^TM^ Fixation/Permeabilization kit (BD Biosciences, #554714) at room temperature for 15 minutes, followed by a briefly rinse with wash buffer. Fixed cells were incubated overnight at 4°C with a primary anti-cathepsin B antibody (abcam). After two washes, an Alexa Fluor 647-conjugated anti-mouse secondary antibody was applied according to the manufacturer’s protocol. Following three additional washes, cells were mounted using fluorescence mounting medium for immunofluorescence analysis. Alexa Fluor 647 fluorescence intensity was evaluated with a Zeiss LSM 700 confocal microscope and BD FACsLyric flow cytometer.

**Lysosome vacuolization imaging**

5.0×10^4^ Raji cells were seeded in a 96-well plate and treated with 50 nM Apilimod, 50 μM OSI-027, 100 nM Rapamycin, and 50 μM BAY-1797 for overnight. Cells were stained with 100 nM LT Deep Red for 30 min. Cells were centrifuged and resuspended in 4 μl fluorescent mounting media on a cover glass visualized under a Zeiss LSM 700 microscope. Vacuoles are shown as red spots.

**Statistical analysis**

Statistical analyses were performed using GraphPad Prism v8. Data are presented as the means ± standard deviation as indicated in the figure legends. For comparisons between two groups, the Mann–Whitney U test was used. For multiple group comparisons, one-way analysis of variance (ANOVA) followed by appropriate post hoc tests was applied. Significance is indicated as *p < 0.05, **p < 0.01, ***p < 0.001, ****p < 0.0001.
